# *Couples Sharing Sleep and Sickness*: Dyadic Sleep‐Wake Patterns, Health Consequences, and Intervention Efficacy in Cancer Patient‐Caregiver Dyads—A Systematic Review and Preliminary Meta‐Analysis

**DOI:** 10.1002/pon.70480

**Published:** 2026-05-11

**Authors:** Jie Zhong, Wei Liang, Mu‐Hsing Ho, Wenjuan Zhao, Chia‐Chin Lin

**Affiliations:** ^1^ School of Nursing LKS Faculty of Medicine The University of Hong Kong Hong Kong China; ^2^ School of Nursing Nanjing Medical University Nanjing China; ^3^ Department of Nursing Shanghai Cancer Center Fudan University Shanghai China; ^4^ Department of Oncology Shanghai Medical College Fudan University Shanghai China

**Keywords:** cancer, couples, meta analysis, sleep disturbances

## Abstract

**Background:**

Sleep disturbances are prevalent among cancer patient‐caregiver dyads, yet their interdependent sleep patterns remain understudied. Therefore, this systematic review aimed to synthesize evidence on dyadic sleep‐wake patterns, health consequences (physical, psychological, and relationship health), and the effectiveness of dyadic intervention (intervention delivered to both members in the dyad) on sleep outcomes in cancer patient‐caregiver pairs.

**Methods:**

Following JBI guidelines, we searched five databases (Cochrane Library, PubMed, CINAHL, Web of Science, EMBASE) for studies assessing dyadic sleep patterns, health outcomes, and interventions in adult cancer dyads. Quality of observational studies was evaluated using the JBI critical appraisal tool for analytical cross‐sectional studies. Quality of randomized controlled trials (RCTs) was evaluated using the Cochrane Collaboration's Tool for Assessing Risk of Bias Version 2.0. Narrative synthesis addressed: (1) dyadic sleep‐wake patterns, (2) health consequences, and (3) sleep outcomes measured in dyadic interventions. Random‐effects meta‐analyses pooled sleep outcomes from interventions.

**Results:**

From 3862 screened studies, 28 were included (19 observational, 9 interventional). Our synthesis of observational studies revealed that sleep disturbances were highly prevalent and co‐occurring within dyads, with members showing moderate‐to‐strong concordance in sleep quality (*r* = 0.31 to 0.68, median *r* = 0.45). Actor effects (one's own sleep affecting one's own health) were consistently strong across physical health (*β* = 0.22–0.57), psychological health (*β* = 0.22–0.57), and relationship health (*β* = 0.03–0.24). Partner effects (one partner's sleep affecting the other's health) ranged from *β* = 0.15 to 0.48 across physical and psychological outcomes. For dyadic interventions, mindfulness‐based approaches were most tested, though family resilience and acceptance/commitment therapies also showed promise. However, only one study explicitly targeted sleep with a dyadic adaptation of Cognitive Behavioral Therapy for Insomnia. A meta‐analysis of nine studies (128 patient participants) showed a small statistically significant effect (SMD = −0.33, 95% CI = −0.62 to −0.04, *p* = 0.02). However, when limiting the analysis to three RCTs with 74 couples, dyadic interventions yielded a non‐significant effect on decreasing patients' sleep disturbances (SMD = −0.22, 95% CI = −0.56 to 0.12). For caregivers, the full meta‐analysis showed a moderate effect (SMD = −0.42, 95% CI = −0.68 to −0.16, *p* = 0.002), and the RCT‐only analysis remained significant but attenuated (SMD = −0.36, 95% CI = −0.71 to −0.02, *p* = 0.04).

**Conclusion:**

This review acknowledged the interdependent nature of sleep among cancer patients and caregivers. Our results could have significant implications in sleep management for healthcare professionals working with dyads impacted by cancer, particularly bed‐sharing couples. Since limited RCTs examined the effectiveness of dyadic interventions on sleep promotion in cancer dyads, further research needs to provide more rigorous evidence in this population.

## 1 Introduction

Optimal sleep is characterized by adequate sleep duration (7–8 h) that is easily initiated (sleep onset latency [SOL] < 20 min) and maintained without disruptions (wake after sleep onset [WASO] < 20 min) [1]. A cancer diagnosis profoundly disrupts physiological, psychological, and physical wellbeing in both patients and their caregivers. Notably, 60.7% of cancer patients across all cancer types and disease stages experience sleep disturbance [2], manifesting as difficulties falling asleep, poor sleep maintenance, suboptimal timing, reduced efficiency, and excessive daytime sleepiness [3, 4]. Objective and subjective measures consistently demonstrate non‐optimal sleep patterns in this population. Short sleep durations, prolonged WASO, and longer SOL have been self‐reported and/or actigraphy‐measured among cancer patients [5, 6, 7, 8, 9, 10] and caregivers [11, 12]. Further, cancer patients with poor sleep are more likely to have cancer recurrence, worse prognosis, cognitive decline, and reduced post‐treatment work capacity [2, 13]. Caregivers often experience persistent sleep disruption even after the patient's hospitalization or death, hindering their return to pre‐caregiving routines [14, 15], with increased risks of depression, anxiety, cardiovascular disease, immune dysfunction, and memory impairments [16].

Sleep disturbances in cancer populations arise from multifactorial causes [17, 18, 19]. Spielman's 3P model, which is a well‐established behavioral framework for insomnia, outlines how predisposing, precipitating, and perpetuating factors interact to initiate and sustain sleep disruption [20]. Predisposing factors (baseline vulnerabilities) could include female sex, older age, personality traits, history of sleep problems, and psychological disorders [18, 21]. Precipitating factors (acute triggers) encompass cancer‐related stressors including inflammatory cytokine fluctuations, disrupted circadian rhythms, treatment side effects, hospitalization, psychological distress, and medications [17, 18, 19]. For cancer caregivers, chronic stress, emotional burden such as uncertainty and lack of support, and resultant symptoms such as fatigue, anxiety, depression from cancer caregiving could act as acute triggers [22, 23, 24]. Perpetuating factors (chronic maintenance) involve maladaptive behaviors and cognitions, such as excessive daytime napping and non‐sleep activities in bed [17, 18, 19]. Unfortunately, despite of its high prevalence and negative consequences, sleep disturbance is frequently overlooked in cancer patients and caregivers. Patients, caregivers, and professionals tend to assume sleep problems as a normal and temporary reaction, which is rarely to be included as part of routine screening in oncology care and contributes to the limited referral and usage of psychological interventions or medications for sleep promotion [25, 26].

Sleep is not merely an individual behavior but a dyadic process. Going to sleep and waking up together is one of the most intimate human actions that optimally occurs when one feels sufficiently safe and secure to down‐regulate vigilance and alertness [27]. The dyadic sleep‐wake patterns could be conceptualized using both the individual‐level and the dyadic‐level metrics. Individual‐level interdependence refers to the degree to which partners' sleep parameters (e.g., sleep quality, duration, and timing) are correlated or similar. Individual‐level analysis such as Pearson correlations, within‐couple difference variance, and paired *t*‐tests are commonly used to quantify the interdependence. In healthy couples, evidence has revealed significant interdependence, where partners' sleep quality, duration, and timing show different levels of interdependence [28, 29, 30]. This interdependence is likely driven by shared sleep‐timing behaviors, movement patterns and sleep stages associated with the physical presence of another person in the bed [31, 32]. Dyadic‐level concordance captures a real‐time and dynamic form of interdependence. Sleep and wake concordance has been defined and operationalized as the degree to which partners share the same sleep or wake state at the same time throughout the night [33, 34], which could be an indicator of coregulation within a couple, as a reciprocally maintained physiological process that serves to maintain psychological and biological homeostasis of individuals in a relationship, which potentially influences downstream health outcomes [33]. Evidence showed that higher sleep‐wake concordance in the couples has been linked with longer sleep duration and better sleep quality [35, 36], lower psychological distress [37], lower sleep and wake blood pressure [33], and lower C‐reactive protein values [33]. Therefore, dyadic sleep (e.g., sleep‐wake concordance) may be a one way to characterize the degree to which physiological and behavioral coregulation occurs within couples, potentially influencing downstream health outcomes [33].

Less known, however, is the extent to which adult cancer patients and bed‐partner caregivers experience dyadic sleep disturbance [38]. For a patient in a romantic relationship or marriage, the partner is always the primary source of care and support. According to the interdependence theory, cancer is depicted as a “we‐disease”, as both patients and partners are profoundly affected in the process of adjusting to the disease, with stressors and coping strategies shared within the relational system [39]. In the context of cancer, the intimate dyadic nature of sleep is critically important but uniquely stressed. The dyad must cope with significant, illness‐specific disruptors to normal sleep‐wake patterns. The shared cancer‐related stress, coupled with potential caregiving demands during the night, can lead to maladaptive dyadic sleep‐wake patterns that collectively compromise the health of both partners [40, 41]. By addressing dyadic sleep disruption and promoting supportive, co‐regulatory behaviors, dyadic interventions could effectively break the cycle of mutual sleep disturbance and improve outcomes for both partners. However, the evidence regarding the nature of dyadic sleep‐wake patterns, their health consequences, and the efficacy of dyadic interventions in cancer populations remains limited and has not been systematically synthesized. Therefore, this systematic review aimed to [1] Characterize dyadic sleep‐wake patterns (both individual‐level and dyadic‐level) in cancer patient‐caregiver dyads [2], Examine the effect of dyadic sleep patterns on health outcomes [3], Evaluate the preliminary effects of existing dyadic interventions on sleep outcomes of both members in the dyad.

## 2 Methods

We conducted the systematic review following the JBI Manual for Evidence Synthesis on conducting systematic reviews of effectiveness [42]. This systematic review was reported based on the PRISMA 2020 guideline [43]. The PRISMA checklist was attached in the Supporting Information S1: Appendix 1. This review was previously registered in PROSPERO (CRD420251076257).

### 2.1 Search Strategy

Searches were conducted in five databases, including the Cochrane Library, PubMed, CINAHL Plus, Web of Science, and EMBASE. Three search terms were combined to develop the search strings in different databases (Table 1), including “sleep”, “cancer”, and “partner*”. The search strategy was intentionally broad to capture all relevant dyadic sleep studies in oncology contexts. No time restriction was applied to ensure comprehensive coverage of emerging literature. Final searches were executed in October 2025 by the first author (JZ). A manual search was also performed on the article reference lists. The systematic review management software Covidence was used for the blinding of the study selection process. After removing the duplicate results, titles and abstracts were screened based on the eligibility criteria by two reviewers (JZ and WL). All full texts were screened independently by two reviewers. Any disagreement was resolved through discussion between the two reviewers, and a third reviewer was consulted when needed.

**TABLE 1:** Search strategies across databases search date: October 2025.

Database (records)	Search strings
PubMed (529)	(“Sleep” [Mesh] OR “Sleep Wake Disorders” [Mesh] OR “Sleep Deprivation” [Mesh] OR sleep [TW] OR insomnia [TW] OR “sleep quality” [TW]) AND (“Neoplasms” [Mesh] OR cancer [TW] OR oncolog* [TW] OR tumor* [TW]) AND (“Spouses” [Mesh] OR “Caregivers” [Mesh] OR dyad*[TW] OR couple*[TW] OR partner*[TW])
CINAHL Plus (234)	(MH “Sleep” OR MH “Sleep Disorders” OR MH “Sleep Deprivation” OR AB sleep OR AB sleep OR AB insomnia OR AB “sleep quality”) AND (MH “Neoplasms” OR AB cancer OR AB oncolog* OR AB tumor*) AND (MH “Spouses” OR MH “Caregivers” OR AB dyad* OR AB couple* OR AB partner*)
Web of Science (952)	TS = ((sleep OR insomnia OR “sleep quality” OR “sleep disturbance*”) AND (cancer OR neoplasm* OR oncolog* OR tumor*) AND (dyad* OR couple* OR partner* OR spous* OR caregiver*))
EMBASE (2145)	1. exp sleep/ OR exp sleep disorder/ OR exp sleep deprivation/ OR (sleep OR insomnia OR “sleep quality”).mp. 2. exp neoplasm/ OR (cancer OR oncolog* OR tumor*).mp. 3. exp spouse/ OR exp caregiver/ OR (dyad* OR couple* OR partner*).mp. 4. 1 AND 2 AND 3
Cochrane Reviews (2)	Title Abstract Keyword (sleep OR insomnia OR “sleep quality” OR “sleep disturbance*”) AND (cancer OR neoplasm* OR oncolog* OR tumor*) AND (dyad* OR couple* OR partner* OR spous* OR caregiver*)

### 2.2 Study Eligibility

Studies were included if they met the following criteria [1]: Focused on sleep in adult cancer patients and their caregivers. Specifically, studies were included if the sample was explicitly described as spousal/partner dyads or if spousal/partner caregivers comprised of the majority (> 50%) of the sample based on sample descriptions [2]. Explored the perspectives on dyadic sleep‐wake patterns, health consequences, or dyadic interventions on sleep outcomes among cancer patient‐caregiver dyads. Study design could be [1] observational studies were included if they reported any sleep metrics (both individual‐level and dyadic‐level) for both dyad members, or [2] interventional studies were included if the intervention was delivered to the dyad (both participated) and a sleep outcome was measured in at least one member. Because dyadic sleep interventions are relatively rare in cancer populations, our review did not restrict to sleep‐focused trials (e.g., dyadic CBT‐I) that measured sleep as the primary outcome. Therefore, we also included dyadic psychosocial interventions (e.g., mindfulness) that measured sleep as a secondary outcome.

Studies were excluded if [1] they included only one member of the dyad (either patients or caregivers) [2], they focused on pediatric cancer populations and their parents [3], articles were not published in peer‐reviewed journals (e.g., dissertations, conference abstracts) or not in English.

### 2.3 Data Extraction

Data from all included studies were extracted independently by JZ and checked for accuracy by WL. Adapted from the JBI Data Extraction Form, data was extracted separately for observational studies (study design, study aims, cancer type and stage, participants, sleep parameters, health consequences and measures, statistical analyses, and main findings) and interventional studies (study design, cancer type and stage, participants, intervention group, control group, outcomes and measures, and main findings on sleep outcomes).

### 2.4 Qualitative Evaluation

Quality of observational studies was evaluated using the JBI critical appraisal tool for analytical cross‐sectional studies. Quality of randomized controlled trials (RCTs) was evaluated using the Cochrane Collaboration's Tool for Assessing Risk of Bias Version 2.0. Each domain within the tool was assessed to have a low or high risk of bias. Quality of the pre‐posttest studies was evaluated with the Non‐randomized Studies‐of Interventions Version 2.0 (ROBINS‐I V2) assessment tool. Each domain was judged to have a low, moderate, serious or critical risk of bias. Two reviewers independently evaluated the quality of included studies, and any conflict would be resolved by consultation with a third reviewer when needed. The Cochrane tools were selected as it is specifically designed to evaluate methodological quality in interventional studies using domain‐bases assessment which directly addresses potential bias (e.g., randomization and blinding).

### 2.5 Data Synthesis and Analysis

A narrative synthesis of included studies was conducted based on our three research questions: Dyadic patterns (individual‐level and dyadic‐level), Health consequences (actor and partner effect on physical and psychosocial health), and Dyadic interventions. Meta‐analysis was not conducted on the observational studies due to substantial clinical and methodological heterogeneity, particularly the small number of studies per outcome domain. Specifically, the 19 observational studies varied in cancer types, disease stages, study designs, outcome measures, and effect size metrics. Therefore, we present a narrative synthesis of effect size ranges and medians (including Pearson's r for correlations, standardized *β* for actor and partner effects) to characterize the consistency and variability of findings across the studies. Meta analyses for dyadic interventions on sleep outcomes were conducted based on the extracted data in Review Manager Software (RevMan Version 5.0) from the Cochrane Collaboration. Heterogeneity across studies was tested with *I* ^*2*^ values, which ranged from 0% to 100%, with higher values indicating greater heterogeneity. When *I* ^*2*^ ≥ 50% demonstrates substantial heterogeneity (*p* < 0.05), a random‐effect model was used; otherwise, a fixed‐effect model would be used. A meta‐analysis was performed to determine the pooled effect of the intervention of standardized mean differences (SMD) for continuous outcomes and the odds ratio (OR) for dichotomous outcomes with 95% confidence intervals (CIs), when at least two studies assessed the similar outcome.

## 3 Results

### 3.1 Characteristics of Included Studies

**FIGURE 1:**
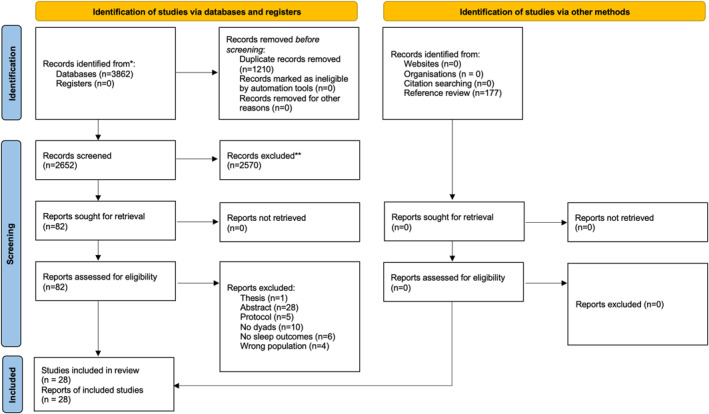
PRISMA flowchart.

The searches yielded 3862 records with 2652 screened for titles and abstracts, and 82 retrieved for full text review. A total of 28 studies were included in the narrative synthesis with19 observational studies [10, 12, 15, 38, 44, 45, 46, 47, 48, 49, 50, 51, 52, 53, 54, 55, 56, 57, 58] and 9 interventional studies [40, 59, 60, 61, 62, 63, 64, 65, 66]. Sample size from the observational studies ranged from 15 to 484 dyads. For interventional studies, there were 3 RCTs with 74 dyads in intervention and 61 dyads in control groups [63, 65, 66] and 6 pretest‐posttest studies with 54 dyads at baseline [40, 59, 60, 61, 62, 64]. Tables 2 and 3 presented the characteristics of observational and interventional studies separately. Most studies were conducted in high‐income countries or regions including US, Germany, UK, Australia, Hong Kong, and Taiwan, except one for mainland China. Cancer type and stage varied across studies including breast [10, 48, 52, 53, 54, 57, 66], lung [47, 59, 60, 61], colorectal [38, 44], gastrointestinal [40, 65], brain [64], or any advanced cancer. The screening process was present in Figure 1.

**TABLE 2:** Characteristics of observational studies.

Author (year)/country	Study design	Study aims	Cancer type and stage	Participants	Sleep parameters	Health impacts	Statistical analyses	Main findings
Diamantis (2025)/US [51]	Secondary analysis of a clinical trial on collaborative care in oncology	To examine the rates as well as the interdependence of depressive symptoms and sleep problems in patients with cancer and their intimate partner family caregivers	A proven cancer diagnosis (via biopsy or radiograph) and at any stage in their treatment (e.g., surveillance, chemotherapy, and surgery)	188 couples Patients: Mean age of 69.3, 56.9% males Caregivers: 63.8, 30.7% 100% married spouses	Subjective sleep quality: PSQI	Depressive symptoms: CES‐D	APIM	Actor effects were observed in which patients' depressive symptoms were associated with shorter sleep duration. No partner effects were observed on the prediction of depressive symptoms on sleep duration
Ellis (2024)/US [49]	Secondary analysis of baseline data from RCT	To identify patient and caregiver symptom clusters and investigate associations between identified clusters and demographic, clinical and psychological factors	Advanced lung, colorectal, breast, or prostate cancer (e.g., stage III or IV)	484 dyads Patients: Mean age of 60.5% and 62% females Caregivers: Mean age of 56.5% and 56.9% females	Sleeping problems: Symptom scale of the omega Screening questionnaire	NA	Latent class analysis was performed to identify symptom clustering in dyads	In the dyads, the high symptom burden subgroup had a higher probability of all symptoms with a high probability (over 75%) of fatigue, mental distress and sleeping problems.
Tsai (2024)/US [44]	Secondary analysis of a larger observational study	To examine the differential associations of cancer‐related posttraumatic stress symptoms (PTSS) with various sleep markers in this population	Colon or rectal cancer (stages I–IV)	138 dyads Patients: Mean age of 56.9, 31.9% females Caregivers: Mean age of 55.3, 68.1% females	SOL, WASO, sleep duration and sleep efficiency: Consensus Sleep diary for 14 consecutive days	Posttraumatic Stress Symptoms (PTSS): Impact of event Scale	APIM	The four PTSS clusters, particularly arousal and reactivity and negative cognitions and mood, have distinct associations with sleep markers individually and dyadically in patients and caregivers.
Ting (2024)/US [38]	A longitudinal study in 7 days	Explore the extent to which sleep indices were associated with physical health and diurnal cortisol patterns among adult patients with cancer and their sleep‐partner caregivers	Colorectal Cancer	81 dyads Patients: Mean age of 55.54, 34.6% females Caregivers: 53.67, 66.7% Mean relationship duration of 23.99 years	Sleep duration, SOL, WASO: Both sleep diary and actigraphy	Physical health: Self‐report PROMIS and an objective biomarker	General linear modeling was employed to test the associations between sleep and physical health markers	Among patients, longer self‐reported WASO was associated with poorer physical health and flatter cortisol slope (*p* ≤ 0.013). Among caregivers, longer self‐reported SOL was associated with poorer physical functioning, actigraphy‐measured WASO was associated with steeper cortisol slope, and longer self‐reported Sleep markers studied than actigraphy‐measured were associated with poorer physical functioning (*p* ≤ 0.042).
Vachon (2024)/US [53]	Secondary analysis from a larger cross‐sectional study	To examine the association of relationship satisfaction concordance between breast cancer survivors (BCSs) and their partners with matched controls on the survivors' physical and psychosocial outcomes	Breast cancer survivors	220 BCSs dyads and 167 matching controls dyads Survivors: 45.3 years	Subjective sleep quality: PSQI	Relationship satisfaction and concordance: ENRICH material Satisfaction	Six separate multiple linear regressions were run for the effect of relationship satisfaction and concordance on each of the dependent variables (including sleep disturbance)	Lower concordance was significantly associated with increased social constraint (*p* = 0.029), increased depression (*p* = 0.038), and increased fatigue (*p* = 0.006). Poor relationship satisfaction and concordance were significantly associated with poor physical and psychosocial outcomes.
Salomo (2024)/Germany [58]	A cross‐sectional study	To quantitatively investigate the dream experiences of oncology patients and explore the interdependence between patients and their partners in terms of dream experiences and life satisfaction	Any types of cancer	101 dyads Patients: Mean age of 64.73 Partners: 63.50	Subjective dream experiences: Jena dream inventory Subjective sleep quality: PSQI	Life satisfaction: Satisfaction with life Scale	APIM	Dream intensity exhibited a significant group‐specific partner effect, but no overall partner effect. For both dyad members significant dreaming predictors for life satisfaction were found.
Kwekkeboom (2024)/US [46]	Cross‐sectional observational Study	To assess and characterize self‐reported symptom cluster experiences in cancer patient‐caregiver dyads	Any types of cancer	30 dyads Patients: Mean age of 62, 79% female Caregivers: Mean age of 60, 38% female	Sleep problems: Symptom severity and distress	NA	Descriptive statistics and graphical display of symptom clusters: Psychoneurological, emotional, and gastrointestinal	Patients most frequently reported experiencing fatigue, sleep problems, dry mouth, anxiety, and pain, with moderate to severe distress. Caregivers most frequently reported experiencing anxiety, sleep problems, feeling angry or irritable, fatigue, and depressed mood, with mild to moderate distress.
Schuler (2023)/Australia [45]	Single‐arm, observational feasibility study	To test wearable sensor (WS)‐triggered ecological momentary assessments (EMAs) and electronic patient‐reported outcomes in community palliative care with patient‐caregiver dyads	Cancer patients undergoing palliative care	15 dyads Patients: Mean age of 50, 20% females Caregivers: Mean age of 59, 73% females	Sleep disturbance: Sleep ecological momentary assessments	NA	Fisher's exact test and a linear mixed‐effect model were used to compare the proportion of recorded EMA severities at the group level (patients vs. caregivers).	Sleep disturbance was similar but for different reasons: Patients (physical symptoms) and caregivers (worrying about the patient).
Fenech (2022)/US [52]	Secondary analysis of a larger longitudinal study	To examine the daily within‐person associations between partner responsiveness and subjective sleep in early‐stage breast cancer survivors and their partners	Early‐stage breast cancer survivors	72 couples Survivors: 57.5 mean age Partners: 59.5 mean age Relationship length: 28 years average	Subjective sleep quality: 2 items from the PSQI	Partner responsiveness: 2 items e.g., “how much did you try to be accepting, understanding, and caring of/toward your partner today?”	APIM	Survivor and partner reports of partner responsiveness were associated with their own subjective sleep. Effects of one participant's partner responsiveness on their partner's sleep were not observed.
Perndorfer (2022)/US [57]	Secondary analysis of a larger longitudinal study	To examine the relationship between fear of recurrence (FCR) and sleep disturbance in breast cancer survivors and their partners	Breast cancer survivors	76 couples Demographics not reported	Sleep duration, sleep quality, SOL, WASO: 21‐day sleep diary bursts	FCR: Fear of cancer recurrence inventory	Structured equation modeling	Survivor FCR was associated with their own reduced sleep duration, reduced sleep quality, and greater sleep onset latency. Survivor FCR was also associated with their partners' reduced sleep quality and greater sleep onset latency. Partner FCR was associated with their own reduced sleep duration, reduced sleep quality, and greater sleep onset latency. Partner FCR was also associated with survivors' reduced sleep quality.
Ellis (2021)/US [48]	Secondary analysis of baseline data from RCT	To investigate symptom prevalence and independent and interdependent associations between symptom distress and quality of life among black american patient/caregiver dyads following a cancer diagnosis	Breast, colorectal, lung, or prostate cancer	151 dyads Patients: Mean age of 59.1, 87% females Caregivers: Mean age of 51.5, 14.9% females	Sleeping problems: Physical distress items	Quality of life: Functional assessment of cancer therapy including physical, social, emotional, and functional wellbeing	APIM and path analysis	Many patients and caregivers have concurrent concerns. 60.3% patients and 53.6% caregivers had sleeping problems. Actor effects: Among patients, increased physical distress was associated with decreased physical, emotional, and functional wellbeing. Among caregivers, increased physical distress was associated with decreased physical and functional wellbeing. Partner effects: Increased patient physical distress was associated with decreased caregiver emotional wellbeing. Increased caregiver physical distress was associated with decreased patient emotional wellbeing but increased patient social wellbeing.
He (2022)/China [47]	Cross‐sectional observational study	To investigate the clinical implications of sleep quality anxiety and depression in patients with advanced lung cancer (LC) and their family caregivers (FCs)	Advanced lung cancer (stage III/IV)	98 dyads Patients: Mean age of 57, 32.7% females Caregivers: 33.9% females	Sleep quality: PSQI with seven components on subjective sleep quality, sleep latency, duration of sleep, sleep efficiency, sleep disturbances, sleep medication usage, and daytime dysfunction	Anxiety and depression status: SAS and SDS	X^2^ test and Pearson's correlation analyses	There were significant associations between the patients and the caregivers scores of PSQI/SAS/SDS. Sleep disturbances in patients and the global PSQI score of caregivers were independent risk factors for patients' first line progression‐free survival. Moreover, patients' sleep latency and epidermal growth factor receptor mutations were significant prognostic factors for their overall survival.
Chen (2020)/US [12]	Prospective observational study	To understand sleep problems and their effects in advanced cancer patients and spousal and intimate partner caregivers and to examine the directionality of the link between patients' and caregivers' sleep problems	Advanced cancers related to the hepatobiliary‐pancreatic system	54 dyads Patients: Mean age of 62, 65% males Caregivers: Mean age of 55, 29% males	Sleep quality: PSQI (sleep quality, sleep latency, sleep duration)	Depression symptoms: CES‐D	Cross‐lagged panel analyses were used to examine relationships in patient and caregivers' sleep quality, latency, and duration over time.	Partners' sleep quality significantly predicted patients' sleep quality from baseline to the 2‐month evaluation. Patients' sleep quality then significantly predicted partners' sleep quality from the 2 months to the 4‐month evaluation. Patients' sleep latency significantly predicted partners' sleep latency from 2 to 4 months. Although sleep duration was highly correlated between patients and partners between baseline to 6‐month, neither patients' nor partners' sleep duration significantly predicted the other's.
Otto (2019)/US [56]	Secondary analysis of a cross‐sectional study	To examine the dyadic impact of anxiety and depression on sleep duration in a sample of advanced cancer patients and their spouse caregivers	Advanced cancer	87 couples Patients: Mean age of 66.75, 71.3% Caregivers: 64.68, 28.7%	Objective sleep duration	Anxiety and depression symptoms: HADS	APIM	Individuals' anxiety was negatively associated with their own and their partner's sleep duration. No significant actor or partner effects were found for depression.
Chan (2017)/Hong kong, China [55]	Secondary analysis of a study examining qigong exercise	Examine the bidirectional effects of anxiety and depression on sleep disturbance in Chinese couples affected by cancer	Any types of cancer	135 couples Patients: Mean age of 58.35, 54.8% males Spouses: 57.88, 45.2% males	Sleep quality: PSQI	Anxiety and depression: HADS	APIM	Both actor and partner effects were significant for the relationship between depression and sleep disturbance.
Kotronoulas (2016)/UK [10]	Observational, repeated measures, dyadic study	To explore changes, similarities, differences and interrelations in the sleep‐wake parameters of patient‐caregiver dyads throughout adjuvant chemotherapy for breast cancer.	Breast cancer undergoing chemotherapy	48 dyads Patients: 100% females Caregivers: 10.4% females	Sleep quality: PSQI	NA	Multivariate hierarchical linear model (MHLM) used for analyzing longitudinal dyadic data. Tau‐correlation coefficients and 95% confidence intervals indicated notable correlations representing the extent of shared variance in each outcome variable for the members of a care dyad.	Most dyads had at least one poor sleeper throughout the study. Curvilinear patterns of change were evident for patients' sleep‐wake parameters, but not in caregivers. Average trajectories were significantly different between the dyad members but indicative of trend for concurrent deterioration at T2. Dyad members' perceived sleep quality, SOL and overall sleep‐wake impairment were closely interrelated.
Hsiao (2014)/Taiwan [54]	A longitudinal study in 8‐month	To explore whether stress from individual's and partner's depression, anxiety, sleep disturbances, insecure attachment and meaning in life were predictors of diurnal cortisol patterns in breast cancer survivors and their spouses	Breast cancer survivors	34 couples Survivors: Mean age of 49.6 Spouses: 53.7	Sleep problems: Medical outcomes Study Sleep Scale (sleep disturbance, snoring, shortness of breath/headache during sleep, sleep adequacy, sleep somnolence and sleep quantity)	Diurnal cortisol patterns	The generalized estimating equations (GEE) model was used to test what were the predictors for the changes in diurnal cortisol patterns across the 8‐month follow‐up period	For survivors and spouses, neither survivors' nor spouses' sleep problems were the predictors of their diurnal cortisol patterns.
Carney (2011)/US [50]	Cross‐sectional observational study, part of a larger longitudinal study	To compare the occurrence rates for and severity ratings of sleep disturbance in patient‐family caregiver (FC) dyads	Four cancer diagnoses (e.g., breast, prostate, lung, brain) undergoing primary or adjunct radiotherapy	102 dyads Patients: Mean age of 64.1, 68.6% males Caregivers: 61.4, 28.4% females	Sleep quality: PSQI and general Sleep disturbance Scale (GSDS) Sleep‐wake parameters: A wrist actigraphy	NA	Differences between dyads for continuous data were evaluated using match‐paired *t* tests. Differences between dyads in categorical data were evaluated using the McNemar test.	Patients with cancer and their FCs experience similar levels of sleep disturbance and that both groups could benefit from interventions that aim to promote restful sleep.
Gibbins (2009)/UK [15]	Prospective, observational, descriptive study	To determine the prevalence of sleep‐wake disturbances in patients with advanced cancer and their carers, to monitor the amount of daytime spent in activity and rest, and to examine the relationship between sleep, physical and psychological symptoms.	Advanced incurable cancer	55 dyads Patients: Mean age of 67, 45% males Carers: Mean age of 66, 55% males	Daytime sleepiness: Epworth Sleepiness Score (ESS) Sleep efficiency (SE), daytime activity, and daytime sleeping: An actiwatch for seven consecutive days	Anxiety and depression: HADS Psychological and physical symptom distress: Memorial Symptom assessment Scale (MSAS) Subjective health status: Short Form‐36	Wilcoxon signed rank test, or the unpaired *t*‐tests was used to compare subjective sleep data of both patients and caregivers. To compare data over the seven consecutive days in either patients or carers alone, ANOVA was used with Bonferroni's multiple comparisons test to determine where significant differences were occurring.	There was no difference between patients and carers who reported having poor sleep or sleep well, in objective of sleep efficiency, sleep fragmentation and daytime activity.

Abbreviations: ANOVA, One‐Way Analysis of Variance; APIM, Actor‐Partner Interdependence Model; CES‐D, Center for Epidemiologic Studies Depression; HADS, Hospital Anxiety and Depression Scale; PSQI, Pittsburgh Sleep Quality Index; RCT, Randomized controlled trial; SAS, Self‐rating Anxiety Scale; SDS, Self‐rating Depression Scale; SOL, Sleep onset latency; WASO, Wake after sleep onset.

**TABLE 3:** Characteristics of interventional studies.

Author (year)/country	Study design	Cancer type and stage	Participants	Intervention group	Control group	Outcome/Measures	Main findings on sleep outcomes
Kim (2024)/US [40]	A single‐arm trial with a pretest/posttest design	Gastrointestinal cancer	10 dyads Patients: 64.53 mean age, 60% females Bed‐partner caregivers: 63.51 mean age, 40% female	My sleep, our Sleep (MSOS): Adaptation of CBT‐I for the significant close relationship nature of sleep and cancer experience Format: Four 1‐h weekly sessions that were delivered via zoom video platform Content: Session 1 (Sleep behavior): Review sleep habits and psychoeducation on sleep hygiene Session 2 (Sleep cognition): Identify noisy thoughts, active mind, automatic negative thoughts, worries, with focus on cancer‐related cognition Session 3 (Sleep cognition): Challenging and reframing noisy thoughts, active mind, automatic negative thoughts, worries, with focus on cancer‐related cognition Session 4 (Sleep in relationship): Effective communication; behaviors, thoughts, and emotions in the cancer journey	NA	Daily sleep assessment in 7 days: Sleep efficiency Subjective sleep quality by the Pittsburgh Sleep quality index	Sleep efficiency of both patients and caregivers, significantly improved after the MSOS intervention (Cohen's *d* = 1.04 and 1.47 for patients and caregivers, respectively, *t* > 3.28, *p* < 0.01). Caregivers' subjective sleep quality also significantly improved (Cohen's *d* = 0.78, *t* = 2.45, *p* = 0.037)
Mosher (2024)/US [63]	RCT	Advanced cancer	33 dyads in the intervention and 22 dyads in the control Intervention: Patients: 61% female, 70.6 mean age, 24% breast cancer and 12% prostate cancer Caregivers: 55% female, 66.7 mean age, 67% spouse or partner, Control: Patients: 64% female, 71.25 mean age, 36% breast cancer and 9% prostate cancer Caregivers: 68% female, 65.8 mean age, 59% spouse or partner	Group‐based mindfulness to enhance quality of life and Support advance care planning (MEANING): Format: Six weekly 2‐h in‐person sessions led by one of two doctoral‐level, certified mindfulness teachers Content: Adapted from mindfulness‐based Stress reduction, interpersonal mindfulness programs and mediation trainings Session 1: Awareness: Meeting ourselves where we are in honesty and kindness Session 2: Perception and creative responding: Wholeness no matter what is here Session 3: Relational presence: Mindful communication skills and hospitality toward the self Session 4: Mindful communication: Cultivating compassion in speech and action; advance care planning as empowerment Session 5: Mindful communication amid challenging thoughts and feelings Session 6: The rest of your life: Making the practice your own	Usual care from their oncology team	Baseline, post‐intervention, and 1 month after intervention Patient quality of life by the McGill quality of life questionnaire Caregiver quality of life by the caregiver quality of life index‐cancer Patient self‐efficacy and readiness for advanced care planning Caregiver burden by the zarit burden interview Patient's and caregiver's symptom and coping measures (PHQ‐8, GAD‐7, PROMIS Sleep disturbance form, mini‐MAC, and PEACE measure)	Multilevel models (MLMs) for dyadic data showed no interaction effects among study condition, time, and role for sleep disturbance.
Rhudy (2023)/US [64]	Pretest and posttest observational pilot study	Brain tumor or stroke	8 dyads Survivors: 37.5% female, 61.0 mean age Caregivers: 100% female, 52.6 mean age, 62.5% spouse or partner	The resilient living program: Tailored for patients with chronic illness and caregivers based on a parent Stress management and resiliency training (SMART) program Format: One, one‐hour telehealth session and online video modules with 5.5 h of content over 8 weeks Content: Not reported	NA	Baseline, 12 weeks and 6 months after intervention Resilience by the connor davidson resilience Scale Stress by perceived Stress Scale Dyadic coping by dyadic coping inventory Quality of life by SF‐36 Symptoms by PROMIS fatigue, anxiety, physical function, and sleep disturbance Caregivers only: caregiver role overload	There were no significant improvements in PROMIS sleep disturbances for both survivors and caregivers.
Burns (2023)/US [65]	Secondary analysis of symptom outcomes from a pilot RCT	Advanced gastrointestinal cancer	20 and 20 dyads in the intervention and control groups Combined: Patients: 55% female, 58.55 mean age Caregivers: 75% female, 52.08 mean age, 62.5% spouse or partner	Telephone‐based, dyadic acceptance and commitment therapy (ACT) Format: Six weekly 50‐min phone sessions delivered by a master's level mental health clinician or a doctoral level psychologist Content: Session 1 (dyadic): Review acceptance‐based coping strategies, and mindfulness Session 2 (individual): Practice mindfulness (awareness of the breath) with therapist Session 3 (individual): Discuss how attempts to avoid thoughts, feelings and symptoms lead to actions not aligned with values and decreased quality of life Session 4 (dyadic): Practice mindfulness and introduce concept of willingness Session 5 (dyadic): Exercises to cultivate a transcendent sense of self from which to accept changing experiences Session 6 (dyadic): Goal setting and review skills	Attention control with education support with six phone sessions	Baseline, 2 weeks and 3 months after intervention Patient and caregiver symptom outcomes: PROMIS measures on sleep disturbance, cognitive concerns, anxiety, depression, and fatigue	Study group differences in symptom outcomes were not statistically significant. However, when examining within‐group change, ACT caregivers experienced moderate reductions in sleep disturbance (effect size = −0.56; −0.49).
Johns (2020)/US [62]	A single‐arm pilot study	Metastatic cancer	13 dyads Patients: 62.91 mean age, 46.15% female Family caregivers: 56.58 mean age, 76.92% female	Mindfully optimizing delivery of end‐of‐life care (MODEL): Format: Two cohorts of 6–7 dyads met as a group for 6 weekly, 2‐h experiential sessions led by a certified mindfulness facilitator Content: Modeled after the mindfulness‐based Stress reduction program with mediation trainings	NA	Baseline, post‐intervention, and 1 month later Advance care planning engagement Quality of life by the McGill quality of life inventory Avoidant coping by the Mini‐MAC Distress by PHQ‐9 and GAD‐7 Sleep disturbance by the Pittsburgh Sleep quality index Fatigue interference by the fatigue Symptom inventory	Both patients and FCGs reported notable reductions in sleep disturbance at 1 month follow‐up.
Milbury (2018)/US [59]	A single‐arm trial with a simple pretest/posttest design	Metastatic non‐small cell lung cancer	6 couples Patients: Mean age of 55.20, 2 females Partners: Mean age of 59.4, 4 females Mean length of marriage of 19.50 years	Couple‐based mind‐body intervention: Format: Couples attended each 60‐min session (four sessions over 2 weeks) with the instructor and daily homework assignment Content: Session 1 (mindfulness): Via education and experiential exercises, couples were taught the three aspects of mindfulness: Being in the present moment, avoiding judgment, and being intentional. Session 2 (connection): Couples were introduced to the idea of connection and loving‐kindness to create positive emotions within themselves and their relationships. Session 3 (gratitude): Couples were prompted to reflect on things, events, and people for which they are grateful via a gratitude meditation followed by an emotional sharing exercise. Session 4 (purpose): Couples brainstormed together to devise strategies to ensure that their lives reflect their self‐identified values and then discussed their ideas with the interventionist guiding this process	NA	Spiritual well‐being using the functional assessment of cancer therapy‐spiritual well‐being Scale Psychological distress using the center for epidemiologic Studies depression Scale Sleep disturbances using the Pittsburgh Sleep quality index	Patients ‐ a large effect for reduced sleep disturbances (*p* = 0.04; *d* = 1.83) Partners ‐ a small effect for increased sleep disturbances (*d* = 0.27)
Hsiao (2016)/Taiwan [66]	RCT	Breast cancer survivors	21 couples in intervention group and 19 in control group Intervention group: Survivors: Mean age of 52.5 Partners: Mean age of 54.1 Control group: Survivors: 47.9 Partners: 50.4	Couples support group: Mindfulness added to family resilience Format: Small group sessions (three to four couples per group) for 120 min every week for 2 months Content: Theme 1: Understand the interrelations between body, mind, and spirit Theme 2: Understand the personal strength and the nature of loss and gain in the life Theme 3: Understand “inner self” as a source of disturbed mind and facilitate self‐acceptance and self‐love to nurture inner self Theme 4: Reconstruct the meaning of life	Usual care	Individual well‐being (sleeping distress, depression, anxiety, meaning of life, quality of life) Relational well‐being (attachment styles in close relationships) Bodily stress responses (cortisol levels)	During the 14‐month study period, there was no significant effect on sleep problem (*p* > 0.05, *χ*2 = 0.886) in the effect of the group‐by‐time interaction.
Milbury (2015)/US [61]	A single‐arm pilot study	Non‐small cell lung cancer stages I through IIIB	10 dyads Patients: 35.7% female, 71.22 mean age Caregivers: 64.3% female, 68.77 mean age, 90% spouse	Couple‐based vivekananda yoga (VKC) intervention Format: 2 to 3 weekly sessions (45–60 min each) over the course of 5–6 weeks Content: (1) deep breathing awareness with visualization; (2) breath retention exercises (e.g., 4‐part breath) (3) mindfulness and focused attention through guided meditation; (4) tsa lung movements; and (5) a brief compassion‐based meditation.	NA	Baseline and post‐intervention Psychological distress by the CESD measure and the anxiety from the brief Symptom inventory Sleep disturbances by the Pittsburgh Sleep quality index Quality of life by the medical outcomes Study Spiritual wellbeing by the functional assessment of cancer therapy spiritual well‐being Scale	For patients, paired t‐ tests revealed a medium effect size for improvements in sleep disturbances (*d* = 0.60). For caregivers, there was a significant reduction in sleep disturbances (*d* = 0.71).
Milbury (2015)/US [60]	A single‐arm pilot study	Non‐small cell lung cancer stages I through IIIB	15 dyads Patients: 44.4% female, 62.16 mean age Caregivers: 66.7% female, 58.95 mean age, 66.7% spouse	Couple‐based vivekananda yoga (VKC) intervention: The same as above	NA	Baseline and post‐intervention	For patients, there was significant improvement in sleep disturbances (*d* = 0.36). For caregivers, there was a significant reduction in sleep disturbances (*d* = 1.01).

Abbreviations: CESD, Epidemiological Studies‐Depression; GAD‐7, Generalized Anxiety Disorder scale; Mini‐MAC, Mini‐Mental Adjustment to Cancer Scale; PEACE, Peaceful; Equanimity, and Acceptance in the Cancer Experience measure; PHQ‐8, Patient Health Questionnaire depression scale; PROMIS, Patient‐Reported Outcomes Measurement Information System.

### 3.2 Quality Appraisal of Included Studies

Results of quality appraisal were shown in Supporting Information S2: Appendix 2. Majority of included observational studies reported clear inclusion criteria, study subjects and settings, predictors and outcomes measures, definition disease condition and controlling for confounding variables [10, 12, 15, 38, 44, 46, 47, 48, 49, 50, 51, 52, 53, 54, 56, 57]. For three RCTs, one RCT showed high quality with low risk of bias in all domains [65] while the other two showed some concerns in blinding of outcome assessors [63, 66]. For non‐randomized studies of interventions, all six studies showed moderate risk without controlling time‐varying covariates from baseline to follow‐up [40, 59, 60, 61, 62, 64], and five studies showed moderate to serious risk due to high attrition rate after intervention with or without comparing the differences between dyads who stayed and withdrew [40, 59, 60, 61, 64]. The high attrition rate might show the difficulty of recruiting and maintaining couples or dyads in the dyadic interventions.

### 3.3 Dyadic Sleep‐Wake Patterns

All of included observational studies used individual‐level sleep metrics (e.g., Pearson correlation between patient and caregiver scores, within‐couple difference scores, paired *t*‐tests) to describe dyadic sleep‐wake patterns among cancer patient‐caregiver dyads. No studies reported the use of dyadic‐level sleep metrics (e.g., sleep concordance, wake transmission, matched chronotype) that would directly capture moment‐to‐moment interdependence or bidirectional influence during shared sleep episodes.

First, con‐occurrence of sleep problems were described. Descriptive analyses indicated that sleep problems were highly prevalent and co‐occurring for both members of the dyad, often embedded within a cluster of other symptoms like fatigue, anxiety, and depression. Two observational studies indicated that concurrent sleep problems affected 60.3% of patients and 53.6% of caregivers [48] or 47% of patients and 42% of caregivers [15]. The reasons for sleep problems might be different in patients (physical symptoms such as pain) and caregivers (worrying about the patient or being disturbed by the patient) [15, 45].

Second, statistical analyses (including Pearson correlation, paired *t*‐test, McNemar test, and one‐way analysis of variance) consistently demonstrated significant associations between patient and caregiver sleep metrics. Patients and caregivers showed strong correlations in key sleep parameters, including sleep duration, sleep onset latency (SOL), wake after sleep onset (WASO) [10, 38], and overall sleep quality (indicated by the Pittsburgh Sleep Quality Index [PSQI]) [10, 47, 55]. For dyadic sleep interdependence on sleep quality, the correlation coefficients were reported cross studies, ranging from *r* = 0.31 to 0.68 (median *r* = 0.45) [10, 47, 55]. Paired tests showed comparable levels of self‐reported sleep duration and SOL, and actigraphy‐measured adjusted sleep duration, SOL, and WASO [38, 50]. Further comparisons revealed that patients and caregivers experienced comparable levels in terms of the occurrence rates for clinically significant levels of sleep disturbance with all three measures ‐ PSQI global score, General Sleep Disturbance Scale (GSDS) score, and total sleep duration [50].

Lastly, differences in some sleep metrics existed between patients and caregivers. Patients typically exhibited more sleep fragmentation [15], longer wake period at night [38], and lower sleep efficiency [50]. In contrast, caregivers demonstrated greater daytime activity, less daytime immobility, and fewer naps [15]. Longitudinal data revealed that while caregivers' sleep patterns may be more stable, both members experience concurrent deterioration at certain points, and the patient's sleep often follows a more variable trajectory [10].

### 3.4 The Effect of Dyadic Sleep Patterns on Health Outcomes

Since no included studies used dyadic sleep metrics, the effect of dyadic sleep patterns on health outcomes focused on individual‐level sleep metrics. The application of the Actor‐Partner Interdependence Model (APIM) across studies revealed complex bidirectional links between one partner's sleep and the health of both individuals within cancer patient‐caregiver dyads. Actor effects (one's own sleep affecting their own health) were consistently strong, while partner effects (one's sleep affecting the other's health) were significant in specific domains, demonstrating the shared health burden.

#### 3.4.1 Physical Health

Sleep disturbances in one partner affected both individual's physical health based on the findings from three observational studies. For patients, poorer sleep (e.g., prolonged WASO and longer SOL) was directly linked to worse physical functioning [38], dysregulated stress physiology (flattened cortisol rhythms) [38], and even shorter progression‐free and overall survival [47]. For caregivers, their own sleep problems (e.g., longer SOL) similarly impaired their physical functioning. Notably, one partner's sleep disruption or psychological state (e.g., anxious attachment) could also dysregulate their partner's stress physiology, demonstrating a direct biological partner effect [54]. Partner effects ranged from *β* = 0.15 to 0.48 (median *β* = 0.29) [47, 51, 54, 55], indicating small‐to‐moderate cross‐partner associations between sleep disruption or psychological distress and biological or functional health indicators.

#### 3.4.2 Psychological Health

Psychological health indicators included depression and anxiety, fears or trauma symptoms, life satisfaction, and quality of life. Depression demonstrated strong actor and partner effects, linking patients' depressive symptoms to their own and caregivers' poorer sleep efficiency and quality [51, 55]. Anxiety exhibits asymmetric effects: while it reliably predicts one's own sleep disruption (actor effects), its impact on partners varies. Otto (2019) found anxiety reduced both partners' sleep duration [56], whereas Chan (2017) observed no significant partner effects [55], possibly due to cultural differences in emotional expression or caregiving roles. Fear of cancer [52] and post‐traumatic stress symptoms [44] created a dyadic cycle of sleep disruption, operating through both actor and partner effects. One partner's fears or trauma symptoms could directly degrade the other's sleep. For quality of life and life satisfaction, actor effects were robust: one's own sleep disturbance or distressing dream experiences lowered their own quality of life [48] and life satisfaction [58]. Significant partner effects also emerged for quality of life, where one partner's physical distress (including sleep problems) could diminish the other's emotional wellbeing [48]. Actor effects ranged from *β* = 0.22 to 0.57 (median *β* = 0.38) [48, 51, 53, 55], and partner effects ranged from *β* = 0.15 to 0.48 (median *β* = 0.29) [12, 48, 51, 55].

#### 3.4.3 Relationship Health

The quality of the relationship was associated with sleep health. Actor effects were significant: when individuals perceived their partner as more responsive and supportive, they reported better personal sleep quality [52]. Conversely, insecure attachment styles (e.g., avoidance) in a partner were associated with their own increased sleep problems [54]. Direct partner effects (where one's behavior directly caused the other's sleep change) were less commonly found in this relational domain Actor effects for partner responsiveness on own sleep quality ranged from *β* = 0.03 to 0.24 (median *β* = 0.14), with consistent positive associations [52, 53]. Partner effects in this domain were generally non‐significant or not reported.

### 3.5 Dyadic Interventions and Its Effectiveness on Sleep Outcomes

Nine interventional studies (3 RCTs, 6 single‐arm trials) have explored dyadic approaches to improve sleep in cancer patient‐caregiver pairs. Interventions have included dyadic cognitive‐behavioral therapy for insomnia (CBT‐I), mindfulness, resilience training, and acceptance and commitment therapy (ACT). They have been delivered in various formats (in‐person, group, telehealth) and across multiple cancer types (e.g., lung, breast, gastrointestinal, brain tumors). Up to note, none of included study directly compared dyadic versus individual intervention delivery for sleep outcomes.

#### 3.5.1 Dyadic Cognitive Behavioral Therapy for Insomnia

Acknowledging the dyadic nature of sleep and cancer experience, the CBT‐I was adapted for the cancer patient‐partner dyads in the single pilot study. My Sleep Our Sleep (*N* = 12 gastrointestinal cancer dyads) was the only study explicitly targeting sleep. It showed large improvements in sleep efficiency (patients: *d* = 1.04; caregivers: *d* = 1.47) and caregiver‐reported sleep quality (*d* = 0.78) [40].

#### 3.5.2 Dyadic Mindfulness Practice

Dyadic mindfulness practices were the most studied interventions in cancer patient‐caregiver dyads. Pilot studies often show promising medium‐to‐large improvements in sleep disturbance, particularly for patients. However, when tested in more rigorous RCTs, significant group‐level effects on sleep were not sustained. Specifically, five included studies examined dyadic mindfulness‐based interventions [59, 60, 61, 62, 63]. The Mindfulness to Enhance Quality of Life and Support Advance Care Planning (MEANING) intervention was adapted from Mindfulness‐Based Stress Reduction and Interpersonal Mindfulness programs, and featured formal mindfulness meditation training such as gentle hatha yoga and sitting meditation. While a pilot study (*N* = 13 dyads) showed promising reductions in sleep disturbance [58], the subsequent RCT (*N* = 55 dyads) found no significant group‐by‐time effects for sleep disturbance [59]. Similarly, the Milbury's team designed and pilot tested the dyadic mindfulness‐based intervention in patients with metastatic lung cancer undergoing treatment and their spousal caregivers [59, 60, 61]. The Couple‐Based Mind‐Body (CBMB) intervention was based on the Mindfulness‐Based Breathing Exercises and Compassion‐Based Mediations. The single‐arm pretest‐posttest study in 4 couples revealed a large effect for reduced sleep disturbance for patients (*d* = 1.83) but not for partners [59]. Two pilot studies of Couple‐Based Vivekanand Yoga (VKC) demonstrated medium effects for both patients (*d* = 0.36–0.60) and caregivers (*d* = 0.71–1.01) [61]. Except for the above five studies, two additional studies incorporated joint mindfulness strategies to emphasize relationship and strengthen relational communication to reduce automatic reactions and habits regarding conflicts and improve their relational wellbeing [65, 66].

#### 3.5.3 Dyadic Resilience Training

Interventions focusing on family resilience have shown minimal direct impact on sleep outcomes [64, 66]. Walsh's family therapy focuses on enhancing family resilience by empowering family belief systems (develop a positive view of living with cancer as an opportunity for personal and family growth), family resources (identify couples' inner strengths, such as hope, courage, responsibility, and determination for a better life for their family), and communication processes (facilitate mutual support and enhance their relational resilience) [66]. The Hsiao's RCT examined the effect of the mindfulness‐incorporated, family resilience‐oriented couples support group in breast cancer survivors and their partners. The couples support group was provided conducted in small group sessions (3–4 couples per group) for 120 min every week for 2 months. The RCT (*N* = 40 dyads) showed reduced anxiety (*χ* ^*2*^ = 20.34, *p* < 0.001) but not sleep problems (*χ* ^*2*^ = 0.886, *p* = 0.927) [66]. The Rhudy's Resilient Living Program was a psychosocial intervention based on a Stress Management and Resiliency Training (SMART) program for parents and tailored to the unique needs of patients with chronic illness and their family caregivers. The intervention consisted of 1, 1‐h telehealth session and 5.5 h of online [64]. The pilot study in a sample of 8 brain tumor/stroke dyads showed no significant sleep improvements at 12 weeks and 6 months [64].

#### 3.5.4 Dyadic Acceptance and Commitment Therapy

The ACT intervention was adapted to patient‐caregiver dyads by incorporating joint mindfulness practices and emphasizing their relationship. The 6‐week phone‐based dyadic ACT program for gastrointestinal cancer dyads (*N* = 40) led to moderate sleep improvements in caregivers but not patients [65].

### 3.6 Meta‐Analysis of Dyadic Intervention Effects on Sleep Outcomes

**FIGURE 2:**
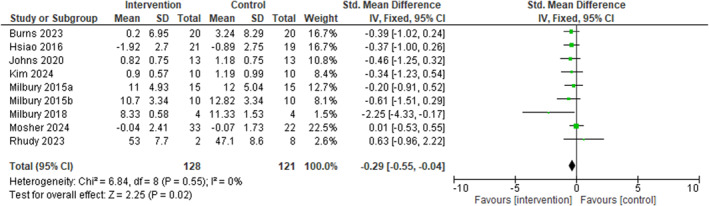
Forest plot on patient's sleep disturbance using 9 interventional studies.

**FIGURE 3:**
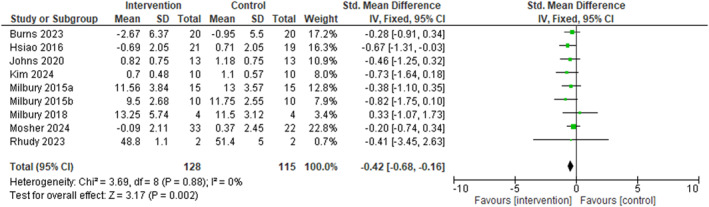
Forest plot on caregiver's sleep disturbance using 9 interventional studies.

**FIGURE 4:**
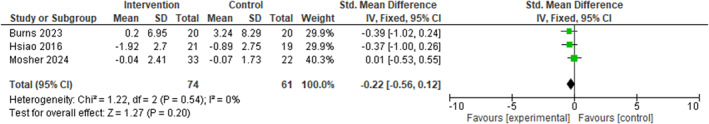
Forest plot on patient's sleep disturbance using 3 RCTs.

**FIGURE 5:**
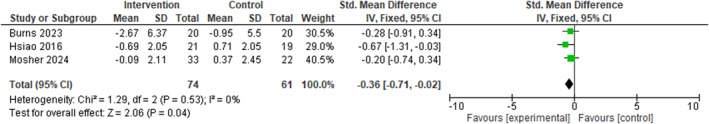
Forest plot on caregiver's sleep disturbance using 3 RCTs.

A meta‐analysis of nine studies (128 patient participants) examined the effect of dyadic interventions on patient sleep disturbance [40, 59, 60, 61, 62, 63, 64, 65, 66] (Figure 2). The pooled effect size showed a small but statistically significant improvement favoring the intervention over control conditions (standardized mean difference [SMD] = −0.33, 95% confidence interval [CI] = −0.62 to −0.04, *p* = 0.02). Heterogeneity was low across studies (I^2^ = 0%, *χ* ^2^ = 6.84, df = 8, *p* = 0.55). A meta‐analysis of nine studies (128 caregiver participants) examined the effect of dyadic interventions on caregiver sleep disturbance [40, 59, 60, 61, 62, 63, 64, 65, 66] (Figure 3). The pooled effect size showed a moderate and statistically significant improvement favoring the intervention over control conditions (SMD = −0.42, 95% CI = −0.68 to −0.16, *p* = 0.002). Heterogeneity was also low (I^2^ = 0%, *χ* ^2^ = 3.69, df = 8, *p* = 0.88). Additionally, when limiting the analysis to three RCTs with 74 dyads, dyadic interventions yielded a non‐significant effect on decreasing patients' sleep disturbances (SMD = −0.22, 95%CI [−0.56, 0.12]) with a low level of heterogeneity indicated by *I* ^*2*^ = 0% (Figure 4) but still a significant effect on decreasing partners' sleep disturbances (SMD = −0.36, 95%CI [−0.71, −0.02]) with a low heterogeneity indicated by *I* ^*2*^ = 0% (Figure 5).

## 4 Discussion

### 4.1 Principle Findings

This systematic review provided the first comprehensive synthesis of evidence on dyadic sleep‐wake patterns, actor‐partner effects linking sleep with health outcomes, and the efficacy of dyadic interventions for sleep outcomes in cancer patient‐caregiver dyads. Our findings underscored the bidirectional interdependence of sleep and health within these dyads, while highlighting critical gaps in intervention research. The current meta‐analyses provide promising but preliminary evidence that nine distinct dyadic interventions demonstrate efficacy in ameliorating sleep disturbances for both cancer patients and their caregivers. However, when limiting analysis to three RCTs with only 74 dyads, dyadic interventions yielded a non‐significant effect on decreasing patients' sleep disturbances, and the RCT‐only analysis remained significant but attenuated in caregivers' sleep disturbance. Thus, current RCT evidence does not support a definitive conclusion that dyadic interventions improve sleep outcomes for patients. The significant pooled effects observed when including single‐arm pre‐post studies (*n* = 9) should be interpreted as preliminary and likely biased by lack of control groups.

In our synthesis of observational studies, two studies reported dyadic sleep‐physical health linkages, including associations with physical functioning, diurnal cortisol rhythms, and patient survival rates [38, 47]. Two studies demonstrated that patients' depressive symptoms negatively impacted their own and caregivers' sleep efficiency and quality (actor and partner effects), suggesting shared emotional burdens [51, 55]. While patients' anxiety consistently predicted their own reduced sleep duration (actor effects), its cross‐partner effects were heterogeneous, possibly due to cultural differences in caregiving roles or emotional expression [55, 56]. The significance of sleep was further linked to life satisfaction [58], fear of cancer recurrence [57], posttraumatic stress [44], quality of life [48], partner responsiveness [52], and insecure attachment [54]. A recent umbrella review confirmed dyadic interventions improve sexual functioning, body image, and dyadic adjustment in cancer dyads [67]. However, effects on psychological outcomes (e.g., distress, anxiety, depression, posttraumatic growth, and fear of cancer recurrence) remain inconsistent [67]. Our review extends this by proposing sleep as a moderating factor. Sleep disturbances may undermine dyadic interventions by exacerbating emotional dysregulation during daytime. Conversely, improving dyadic sleep could facilitate psychological adaptation to cancer. This aligns with high‐quality evidence showing that insomnia interventions reduce both sleep problems and comorbid psychological symptoms, supporting that sleep problems could be a contributory causal factor in the occurrence of psychological problems [68]. Therefore, our results uniquely highlighted the significance of screening and managing sleep disturbance in maintaining health and wellbeing among cancer dyads.

Crucially, our review revealed that among the various therapeutic approaches examined, the most tested dyadic approaches with sleep benefits in oncology care were mindfulness‐based interventions [59, 60, 61, 62, 63]. Mindfulness, rooted in cultivating the compassionate acceptance of present‐moment experiences, is designed to increase distress intolerance or acceptance of unpleasant thoughts and feelings [63]. Its application proved particularly valuable in two critical contexts: facilitating difficult end‐of‐life conversations in advanced cancer dyads [62, 63] and addressing the interconnected psychospiritual needs of couples navigating cancer treatment [59, 60, 61]. The success of mindfulness approaches likely stems from their ability to mitigate the shared emotional burden that frequently underlies dyadic sleep disturbances. Secondly, psychological interventions incorporating family resilience therapy [64, 66] and acceptance and commitment therapy [65] represented another important therapeutic avenue. These approaches guided couples in reframing cancer‐related stress as an opportunity for personal and relational growth, fostering adaptive coping mechanisms that may indirectly improve sleep quality through reduced nighttime rumination and hyperarousal. Lastly, our synthesis revealed an important gap that only one identified intervention specifically targeted sleep behavior changes as its primary outcome [40]. This dyadic CBT‐I program departed from broader psychosocial approaches by employing focused sleep psychoeducation, cognitive restructuring of maladaptive sleep beliefs, and dyadic communication training to reinforce healthy sleep maintenance and prevent relapse. The strong rationale for dyadic sleep interdependence, combined with the established efficacy of individual CBT‐I in oncology care, creates a compelling argument for the future development and rigorous testing of dyadic CBT‐I protocols.

### 4.2 Clinical and Research Implications

This review acknowledged the interdependent nature of sleep in the cancer patient‐caregiver dyads, demonstrating that sleep disturbance is a shared experience with negative effects on both members in the dyad. These findings offer specific implications for both clinical practice and future research aimed at improving dyadic sleep management.

For clinical practice, the findings advocate for a more holistic approach to sleep management within cancer care. Healthcare professionals can make it a routine practice to evaluate the sleep of both the patient and their primary caregiver, especially when they share a bed. By viewing sleep as a shared experience rather than an individual problem, clinicians can unlock new opportunities to support the well‐being of cancer dyads, ultimately leading to more compassionate, effective, and sustainable care. The preliminary evidence suggests that psychosocial interventions delivered to the dyad, such as mindfulness or resilience training, can yield benefits for both partners' sleep. For sleep difficulties due to maladaptive cognitions and behaviors, dyadic CBT‐I could help partners align around healthier sleep habits. Ultimately, sleep disturbance should be integrated as a core component of comprehensive supportive care for dyads, with management plans that address both cancer‐specific factors (e.g., medication side effects) and the couple's shared sleep environment.

The review also highlights several critical pathways for future research. First, to build a more robust evidence base, observational research must adopt longitudinal designs and incorporate objective measures like actigraphy for both partners. Secondly, there is a clear need to develop and implement interventions to target dyadic sleep disruption. Future studies need to rigorously design RCTs that compare the efficacy of a dyadic sleep intervention (e.g., a couple‐based adaptation of CBT‐I) against standard individual care. Importantly, no included study directly compared dyadic versus individual intervention delivery for sleep outcomes. Therefore, whether dyadic approaches confer additional benefit beyond individual interventions remains unknown and should be a priority for future research. Finally, intervention research should strive to elucidate the mechanisms of change (such as whether improvements stem from reduced relationship strain, enhanced emotional co‐regulation, or synchronized healthy routines) to refine the most effective components of dyadic sleep programs.

### 4.3 Strengths and Limitations

This was the first review to comprehensively synthesize evidence on dyadic sleep‐wake patterns and interventions specifically in cancer patient‐caregiver dyads. Our results integrated both observational studies (highlighting dyadic interdependence) and interventional studies (evaluating efficacy), providing a holistic view of the evidence. There are several limitations to be noted. Although meta‐analyses showed the promising effectiveness of dyadic interventions on reducing sleep disturbance in both patients and caregivers, the sample of interventional studies was small and only 3 RCTs were included. More importantly, only one study designed and implemented dyadic intervention that targeted at dyadic sleep behavioral change. Therefore, the positive findings from our meta‐analysis must be interpreted with caution, as the included interventional studies were not primarily designed to target sleep, and non‐significant sleep outcomes in such trials are less likely to be published or reported in detail. Rigorous RCT studies with actigraphy‐based, objective sleep measures are needed as higher level of evidence. Moreover, language limitation in English might exclude studies from non‐English countries. Lastly, we extended our population to family caregivers rather than all spousal caregivers. Although majority of family caregivers from included studies were reported to be spouses or partners, the rest of them could be other family members or friends. Relationship dynamics with partners or family members are different, therefore, future studies focusing on spousal caregivers and their interactions with cancer patients are needed.

## 5 Conclusions

This systematic review highlighted the interdependent nature of sleep in cancer‐caregiver dyads. The included 19 observational studies revealed that sleep disturbances were highly prevalent and co‐occurring within dyads, with members showing moderate‐to‐strong concordance in sleep quality. However, none of included studies used dyadic‐level metrics to capture real‐time sleep interdependence among cancer dyads. For dyadic interventions, current RCT evidence does not support definitive conclusions for patients. For caregivers, the pooled effect was significant but preliminary, requiring cautious interpretation given the small evidence base. Further research with rigorous designs, dyadic‐level sleep measures, and sleep‐focused interventions with comparison of individual interventions is urgently needed.

## Author Contributions

**Jie Zhong:** conceptualization, methodology, investigation (study screening and data extraction), formal analysis (meta‐analysis), writing – original draft, writing – review and editing. **Wei Liang:** investigation (study screening and data extraction), validation, formal analysis (meta‐analysis). **Mu‐Hsing Ho:** investigation (study screening and data extraction), validation, formal analysis (meta‐analysis), writing – review and editing. **Wenjuan Zhao:** validation, writing – review and editing. **Chia‐Chin Lin:** conceptualization, supervision, project administration, writing – review and editing.

## Funding

This work was supported by the Seed Fund for Basic Research for New Staff of The University of Hong Kong.

## Conflicts of Interest

The authors declare no conflicts of interest.

## Supporting information


Supporting Information S1

Supporting Information S2

## Data Availability

Data sharing not applicable to this article as no datasets were generated or analyzed during the current study.

## References

[1] N. F. Watson , M. S. Badr , G. Belenky , et al., “Joint Consensus Statement of the American Academy of Sleep Medicine and Sleep Research Society on the Recommended Amount of Sleep for a Healthy Adult: Methodology and Discussion,” Journal of Clinical Sleep Medicine 11, no. 8 (2015): 931–952, 10.5664/jcsm.4950. 26235159 PMC4513271

[2] M. Al Maqbali , M. Al Sinani , A. Alsayed , and A. M. Gleason , “Prevalence of Sleep Disturbance in Patients With Cancer: A Systematic Review and Meta‐Analysis,” Clinical Nursing Research 31, no. 6 (July 2022): 1107–1123, 10.1177/10547738221092146. 35484919 PMC9266067

[3] A. M. Berger , E. E. Matthews , and M. S. Aloia , “Sleep and Cancer,” in The MASCC Textbook of Cancer Supportive Care and Survivorship (Springer Nature, 2018), 53–65.

[4] J. L. Otte , J. S. Carpenter , S. Manchanda , et al., “Systematic Review of Sleep Disorders in Cancer Patients: Can the Prevalence of Sleep Disorders Be Ascertained?,” Cancer Medicine 4, no. 2 (2015): 183–200, 10.1002/cam4.356. 25449319 PMC4329003

[5] G. E. Dean , N. S. Redeker , Y. J. Wang , et al., “Sleep, Mood, and Quality of Life in Patients Receiving Treatment for Lung Cancer,” Oncology Nursing Forum 40, no. 5 (September 2013): 441–451, 10.1188/13.ONF.441-451. 23989018 PMC4080434

[6] A. Dhruva , K. Lee , S. M. Paul , et al., “Sleep‐Wake Circadian Activity Rhythms and Fatigue in Family Caregivers of Oncology Patients,” Cancer Nursing 35, no. 1 (January 2012): 70–81, 10.1097/NCC.0b013e3182194a25. 21760489 PMC3197878

[7] G. Jakobsen , M. Engstrøm , M. Thronæs , et al., “Sleep Quality in Hospitalized Patients With Advanced Cancer: An Observational Study Using Self‐Reports of Sleep and Actigraphy,” Supportive Care in Cancer 28, no. 4 (April 2020): 2015–2023, 10.1007/s00520-019-04998-5. 31392550

[8] O. Palesh , A. Aldridge‐Gerry , J. M. Zeitzer , et al., “Actigraphy‐Measured Sleep Disruption as a Predictor of Survival Among Women With Advanced Breast Cancer,” Sleep 37, no. 5 (May 2014): 837–842, 10.5665/sleep.3642. 24790261 PMC3985107

[9] J. D. Pawl , S. Y. Lee , P. C. Clark , and P. R. Sherwood , “Sleep Characteristics of Family Caregivers of Individuals With a Primary Malignant Brain Tumor,” Oncology Nursing Forum 40, no. 2 (March 2013): 171–179, 10.1188/13.ONF.171-179. 23448742 PMC3880563

[10] G. Kotronoulas , Y. Wengström , and N. Kearney , “Alterations and Interdependence in Self‐Reported Sleep‐Wake Parameters of Patient–Caregiver Dyads During Adjuvant Chemotherapy for Breast Cancer,” Oncology Nursing Forum 43, no. 3 (May 2016): 288–301, 10.1188/16.ONF.288-301. 27105191

[11] P. A. Carter , “A Brief Behavioral Sleep Intervention for Family Caregivers of Persons With Cancer,” Cancer Nursing 29, no. 2 (April 2006): 95–103, 10.1097/00002820-200603000-00003. 16565618

[12] Q. Chen , L. Terhorst , A. Lowery‐Allison , et al., “Sleep Problems in Advanced Cancer Patients and Their Caregivers: Who Is Disturbing Whom?,” Journal of Behavioral Medicine 43, no. 4 (August 2020): 614–622, 10.1007/s10865-019-00088-3. 31435891 PMC7035154

[13] L. G. Sigurdardottir , U. A. Valdimarsdottir , L. A. Mucci , et al., “Sleep Disruption Among Older Men and Risk of Prostate Cancer,” Cancer Epidemiology, Biomarkers & Prevention 22, no. 5 (May 2013): 872–879, 10.1158/1055-9965.EPI-12-1227-T. PMC3652595 23652374

[14] P. A. Carter , S. Q. Mikan , and C. Simpson , “A Feasibility Study of a Two‐Session Home‐Based Cognitive Behavioral Therapy‐Insomnia Intervention for Bereaved Family Caregivers,” Palliative & Supportive Care 7, no. 2 (June 2009): 197–206, 10.1017/S147895150900025X. 19538802

[15] J. Gibbins , R. McCoubrie , A. H. Kendrick , G. Senior‐Smith , A. N. Davies , and G. W. Hanks , “Sleep‐Wake Disturbances in Patients With Advanced Cancer and Their Family Carers,” Journal of Pain and Symptom Management 38, no. 6 (December 2009): 860–870, 10.1016/j.jpainsymman.2009.04.025. 19800196

[16] K. F. Maltby , C. R. Sanderson , E. A. Lobb , and J. L. Phillips , “Sleep Disturbances in Caregivers of Patients With Advanced Cancer: A Systematic Review,” Palliative & Supportive Care 15, no. 1 (February 2017): 125–140, 10.1017/S1478951516001024. 28095943

[17] M. Kwon , M. V. McPhillips , F. Dong , et al., “Perpetuating Factors of Insomnia in Cancer Survivors,” Oncology Nursing Forum 51, no. 3 (April 2024): 210–222, 10.1188/24.ONF.210-222. 38668908 PMC12969689

[18] D. Howell , T. K. Oliver , S. Keller‐Olaman , et al., “Sleep Disturbance in Adults With Cancer: A Systematic Review of Evidence for Best Practices in Assessment and Management for Clinical Practice,” Annals of Oncology 25, no. 4 (April 2014): 791–800, 10.1093/annonc/mdt506. 24287882

[19] D. Riemann , F. Benz , R. J. Dressle , et al., “Insomnia Disorder: State of the Science and Challenges for the Future,” Journal of Sleep Research 31, no. 4 (August 2022): e13604, 10.1111/jsr.13604. 35460140

[20] A. J. Spielman , L. S. Caruso , and P. B. Glovinsky , “A Behavioral Perspective on Insomnia Treatment,” Psychiatric Clinics of North America 10, no. 4 (December 1987): 541–553, 10.1016/s0193-953x(18)30532-x. 3332317

[21] C. J. Harvey , P. Gehrman , and C. A. Espie , “Who Is Predisposed to Insomnia: A Review of Familial Aggregation, Stress‐Reactivity, Personality and Coping Style,” Sleep Medicine Reviews 18, no. 3 (June 2014): 237–247, 10.1016/j.smrv.2013.11.004. 24480386

[22] G. H. mei , D. mei Chuang , F. Yang , et al., “Prevalence and Determinants of Depression in Caregivers of Cancer Patients: A Systematic Review and Meta‐Analysis,” Medicine 97, no. 39 (September 2018): e11863, 10.1097/MD.0000000000011863. 30278483 PMC6181540

[23] L. Funk , K. Stajduhar , C. Toye , S. Aoun , G. Grande , and C. Todd , “Part 2: Home‐Based Family Caregiving at the End of Life: A Comprehensive Review of Published Qualitative Research (1998‐2008),” Palliative Medicine 24, no. 6 (September 2010): 594–607, 10.1177/0269216310371411. 20576673

[24] Q. Zhang , D. Yao , J. Yang , and Y. Zhou , “Factors Influencing Sleep Disturbances Among Spouse Caregivers of Cancer Patients in Northeast China,” PLoS One 9, no. 10 (October 2014): e108614, 10.1371/journal.pone.0108614. 25275619 PMC4183522

[25] J. R. Davidson , D. Feldman‐Stewart , S. Brennenstuhl , and S. Ram , “How to Provide Insomnia Interventions to People With Cancer: Insights From Patients,” Psycho‐Oncology 16, no. 11 (November 2007): 1028–1038, 10.1002/pon.1183. 17352006

[26] L. Strøm , J. T. Danielsen , A. Amidi , A. L. Cardenas Egusquiza , L. M. Wu , and R. Zachariae , “Sleep During Oncological Treatment – a Systematic Review and Meta‐Analysis of Associations With Treatment Response, Time to Progression and Survival,” Frontiers in Neuroscience 16 (April 2022): 16, 10.3389/fnins.2022.817837. PMC9063131 35516799

[27] K. Richter , S. Adam , L. Geiss , L. Peter , and G. Niklewski , “Two in a Bed: The Influence of Couple Sleeping and Chronotypes on Relationship and Sleep. An Overview,” Chronobiology International 33, no. 10 (November 2016): 1464–1472, 10.1080/07420528.2016.1220388. 27624285 PMC5152533

[28] Y. Fan , H. Chen , J. Lin , Z. Ma , D. Wang , and F. Fan , “Dyadic Associations of Loneliness With Sleep Between Husbands and Wives in Older Couples: Findings From a National 10‐Year Longitudinal Study,” Aging & Mental Health 30 (November 2025): 1–12, 10.1080/13607863.2025.2578182. 41185528

[29] D. Huang , Z. Liu , S. Ma , M. Liu , C. Liu , and A. Liu , “The Relationship Between Night Sleep Duration, Sleep Quality and Depressive Symptoms in Chinese Elderly Couples,” Geriatric Nursing 59 (September 2024): 623–629, 10.1016/j.gerinurse.2024.07.024. 39182443

[30] Y. Wang , X. Guo , Y. Wang , N. Guo , and Q. Yang , “Quality of Relationship, Depression, and Sleep Quality in Patients With Chronic Heart Failure and Their Spouse: An Actor‐Partner Interdependence Model,” BMC Nursing 24, no. 1 (August 2025): 1070, 10.1186/s12912-025-03711-8. 40813687 PMC12351949

[31] T. Elsey , P. S. Keller , and M. El‐Sheikh , “The Role of Couple Sleep Concordance in Sleep Quality: Attachment as a Moderator of Associations,” Journal of Sleep Research 28, no. 5 (October 2019): e12825, 10.1111/jsr.12825. 30790373 PMC6702108

[32] H. E. Gunn , D. J. Buysse , B. P. Hasler , A. Begley , and W. M. Troxel , “Sleep Concordance in Couples Is Associated With Relationship Characteristics,” Sleep 38, no. 6 (June 2015): 933–939, 10.5665/sleep.4744. 25581920 PMC4434560

[33] H. E. Gunn , D. J. Buysse , K. A. Matthews , C. E. Kline , M. R. Cribbet , and W. M. Troxel , “Sleep–Wake Concordance in Couples Is Inversely Associated With Cardiovascular Disease Risk Markers,” Sleep 40, no. 1 (December 2016): zsw028, 10.1093/sleep/zsw028. PMC5968335 28364457

[34] E. M. Walters , A. J. K. Phillips , A. Mellor , et al., “Sleep and Wake Are Shared and Transmitted Between Individuals With Insomnia and Their Bed‐Sharing Partners,” Sleep 43, no. 1 (January 2020): zsz206, 10.1093/sleep/zsz206. 31553049

[35] Z. Tanyi , V. Mészáros , M. Smohai , et al., “Morningness‐Eveningness, Relationship Quality, and Quality of Life Among Couples Living Together,” Chronobiology International 37, no. 12 (December 2020): 1736–1747, 10.1080/07420528.2020.1802289. 32806970

[36] S. J. Wilson and J. R. Novak , “The Implications of being “In it Together”: Relationship Satisfaction and Joint Health Behaviors Predict Better Health and Stronger Concordance Between Partners,” Annals of Behavioral Medicine 56, no. 10 (October 2022): 1014–1025, 10.1093/abm/kaab099. 34849523 PMC9528786

[37] J. H. Chen , “Couples’ Sleep and Psychological Distress: A Dyadic Perspective,” Journals of Gerontology: Serie Bibliographique 73, no. 1 (January 2018): 30–39, 10.1093/geronb/gbx001. 28164225

[38] A. Ting , T. C. Tsai , J. M. Zeitzer , A. Mendez , and Y. Kim , “Sleep Composition of Patients With Colorectal Cancer and Their Sleep‐Partner Caregivers: Physical Health Correlates of Sleep Diary and Actigraphy Measurements,” Psycho‐Oncology 33, no. 8 (2024): e9302, 10.1002/pon.9302. 39123341 PMC11328964

[39] K. Kayser , L. E. Watson , and J. T. Andrade , “Cancer as a “We‐Disease”: Examining the Process of Coping From a Relational Perspective. Families,” Systems, & Health 25, no. 4 (2007): 404–418, 10.1037/1091-7527.25.4.404.

[40] Y. Kim , A. Ting , T. C. Tsai , and C. S. Carver , “Dyadic Sleep Intervention for Adult Patients With Cancer and Their Sleep Partner Caregivers: A Feasibility Study,” Palliative & Supportive Care 22, no. 2 (April 2024): 226–235, 10.1017/S1478951523000627. 37312582 PMC10719417

[41] T. H. Monk , D. J. Buysse , J. M. Potts , J. M. DeGrazia , and D. J. Kupfer , “Morningness‐Eveningness and Lifestyle Regularity,” Chronobiology International 21, no. 3 (January 2004): 435–443, 10.1081/CBI-120038614. 15332448

[42] E. Aromataris , C. Lockwood , K. Porritt , G. Pilla , and Z. Jordan , eds., JBI Manual for Evidence Synthesis (Springer Nature, 2024), https://jbi‐global‐wiki.refined.site/space/MANUAL.

[43] A. Liberati , D. G. Altman , J. Tetzlaff , et al., “The PRISMA Statement for Reporting Systematic Reviews and Meta‐Analyses of Studies That Evaluate Healthcare Interventions: Explanation and Elaboration,” BMJ 339, no. jul21 1 (July 2009): b2700, 10.1136/bmj.b2700. 19622552 PMC2714672

[44] T. C. Tsai , H. R. Mitchell , J. Zeitzer , et al., “Dyadic Investigation of Posttraumatic Stress Symptoms and Daily Sleep Health in Patients With Cancer and Their Caregivers,” Psychosomatic Medicine 86, no. 4 (May 2024): 234–243, 10.1097/PSY.0000000000001283. 38345316 PMC11081839

[45] T. Schuler , C. King , T. Matsveru , et al., “Wearable‐Triggered Ecological Momentary Assessments Are Feasible in People With Advanced Cancer and Their Family Caregivers: Feasibility Study From an Outpatient Palliative Care Clinic at a Cancer Center,” Journal of Palliative Medicine 26, no. 7 (July 2023): 980–985, 10.1089/jpm.2022.0535. 37134212

[46] K. L. Kwekkeboom , J. M. Stevens , A. Berghoff , and K. Litzelman , “Self‐Report of Symptom Cluster Experiences in Cancer patient‐caregiver Dyads,” Supportive Care in Cancer 32, no. 9 (September 2024): 604, 10.1007/s00520-024-08818-3. 39167234 PMC11346582

[47] Y. He , L. Y. Sun , K. W. Peng , et al., “Sleep Quality, Anxiety and Depression in Advanced Lung Cancer: Patients and Caregivers,” BMJ Supportive & Palliative Care 12, no. e2 (July 2022): e194–e200, 10.1136/bmjspcare-2018-001684. 32253349

[48] K. R. Ellis , S. Oh , H. K. Hecht , and L. Northouse , “Symptom Distress and Quality of Life Among Black Americans With Cancer and Their Family Caregivers,” Psycho‐Oncology 30, no. 8 (August 2021): 1356–1365, 10.1002/pon.5691. 33861891 PMC8672379

[49] K. R. Ellis , A. Furgal , F. Wayas , et al., “Symptom Burden and Quality of Life Among Patient and Family Caregiver Dyads in Advanced Cancer,” Quality of Life Research 33, no. 11 (November 2024): 3027–3038, 10.1007/s11136-024-03743-8. 39046614

[50] S. Carney , T. Koetters , M. Cho , et al., “Differences in Sleep Disturbance Parameters Between Oncology Outpatients and Their Family Caregivers,” Journal of Clinical Orthodontics 29, no. 8 (March 2011): 1001–1006, 10.1200/JCO.2010.30.9104. PMC3068050 21282549

[51] G. N. Diamantis , Y. Kim , Z. Ofori‐Atta , et al., “The Interdependence of Depressive Symptoms and Sleep in Dyads Affected by Cancer,” Health Psychology 44, no. 4 (2025): 391–400, 10.1037/hea0001449. 39585770 PMC11932778

[52] A. L. Fenech , C. Perndorfer , E. C. Soriano , et al., “Daily Partner Responsiveness and Everyday Sleep Outcomes in Breast Cancer Survivors and Their Partners,” Supportive Care in Cancer 30, no. 9 (September 2022): 7561–7568, 10.1007/s00520-022-07195-z. 35676343

[53] E. A. Vachon , E. Krueger , D. A. Haggstrom , and V. L. Champion , “The Association Between Relationship Satisfaction Concordance and Breast Cancer Survivors’ Physical and Psychosocial Well‐Being,” Healthcare 12, no. 2 (January 2024): 134, 10.3390/healthcare12020134. 38255023 PMC10815840

[54] F. H. Hsiao , G. M. Jow , W. H. Kuo , et al., “The Partner’s Insecure Attachment, Depression and Psychological Well‐Being as Predictors of Diurnal Cortisol Patterns for Breast Cancer Survivors and Their Spouses,” Stress: The International Journal on the Biology of Stress 17, no. 2 (March 2014): 169–175, 10.3109/10253890.2014.880833. 24393005

[55] J. S. M. Chan , N. X. Yu , A. Y. M. Chow , et al., “Dyadic Associations Between Psychological Distress and Sleep Disturbance Among Chinese Patients With Cancer and Their Spouses,” Psycho‐Oncology 26, no. 6 (2017): 856–861, 10.1002/pon.4240. 27479290

[56] A. K. Otto , B. D. Gonzalez , R. E. Heyman , S. T. Vadaparampil , L. Ellington , and M. Reblin , “Dyadic Effects of Distress on Sleep Duration in Advanced Cancer Patients and Spouse Caregivers,” Psycho‐Oncology 28, no. 12 (December 2019): 2358–2364, 10.1002/pon.5229. 31518026 PMC6898749

[57] C. Perndorfer , E. C. Soriano , S. D. Siegel , R. M. C. Spencer , A. K. Otto , and J. P. Laurenceau , “Fear of Cancer Recurrence and Sleep in Couples Coping With Early‐Stage Breast Cancer,” Annals of Behavioral Medicine 56, no. 11 (November 2022): 1131–1143, 10.1093/abm/kaac018. 35551585 PMC9635995

[58] S. Salomo , T. Hackl , J. Hübner , and B. Hagemeyer , “Dreaming in Patients With Cancer and Their Partners–An Underestimated Factor for Quality of Life?,” Journal of Sleep Research 33, no. 5 (2024): e14169, 10.1111/jsr.14169. 38384003

[59] K. Milbury , R. Engle , A. Tsao , et al., “Pilot Testing of a Brief Couple‐Based Mind‐Body Intervention for Patients With Metastatic Non‐Small Cell Lung Cancer and Their Partners,” Journal of Pain and Symptom Management 55, no. 3 (March 2018): 953–961, 10.1016/j.jpainsymman.2017.11.027. 29208478 PMC6620018

[60] K. Milbury , S. Mallaiah , G. Lopez , et al., “Vivekananda Yoga Program for Patients With Advanced Lung Cancer and Their Family Caregivers,” Integrative Cancer Therapies 14, no. 5 (September 2015): 446–451, 10.1177/1534735415583554. 25917816 PMC4537807

[61] K. Milbury , A. Chaoul , R. Engle , et al., “Couple‐Based Tibetan Yoga Program for Lung Cancer Patients and Their Caregivers: TYC in Lung Cancer,” Psycho‐Oncology 24, no. 1 (January 2015): 117–120, 10.1002/pon.3588. 24890852 PMC4437691

[62] S. A. Johns , K. Beck‐Coon , P. V. Stutz , et al., “Mindfulness Training Supports Quality of Life and Advance Care Planning in Adults With Metastatic Cancer and Their Caregivers: Results of a Pilot Study,” American Journal of Hospice & Palliative Care 37, no. 2 (February 2020): 88–99, 10.1177/1049909119862254. 31378080 PMC8112585

[63] C. E. Mosher , K. A. Beck‐Coon , W. Wu , et al., “Mindfulness to Enhance Quality of Life and Support Advance Care Planning: A Pilot Randomized Controlled Trial for Adults With Advanced Cancer and Their Family Caregivers,” BMC Palliative Care 23, no. 1 (September 2024): 232, 10.1186/s12904-024-01564-7. 39342143 PMC11439323

[64] L. M. Rhudy , E. A. Hines , E. M. Farr , D. Esterov , and S. S. Chesak , “Feasibility and Acceptability of the Resilient Living Program Among Persons With Stroke or Brain Tumor and Their Family Caregivers,” NRE 52, no. 1 (January 2023): 123–135, 10.3233/NRE-220127. 36617758

[65] M. F. Burns , E. Secinti , S. A. Johns , et al., “Impact of Acceptance and Commitment Therapy on Physical and Psychological Symptoms in Advanced Gastrointestinal Cancer Patients and Caregivers: Secondary Results of a Pilot Randomized Trial,” Journal of Contextual Behavioral Science 27 (January 2023): 107–115, 10.1016/j.jcbs.2023.01.001. 37064761 PMC10100868

[66] F. H. Hsiao , G. M. Jow , W. H. Kuo , et al., “The Long‐Term Effects of Mindfulness Added to Family Resilience‐Oriented Couples Support Group on Psychological Well‐Being and Cortisol Responses in Breast Cancer Survivors and Their Partners,” Mindfulness 7, no. 6 (December 2016): 1365–1376, 10.1007/s12671-016-0578-9.

[67] Q. Sun , K. Wang , Y. Chen , X. Peng , X. Jiang , and J. Peng , “Effectiveness of Dyadic Interventions Among Cancer Dyads: An Overview of Systematic Reviews and Meta‐Analyses,” Journal of Clinical Nursing 33, no. 2 (2024): 497–530, 10.1111/jocn.16890. 37876319

[68] D. Freeman , B. Sheaves , G. M. Goodwin , et al., “The Effects of Improving Sleep on Mental Health (OASIS): A Randomised Controlled Trial With Mediation Analysis,” Lancet Psychiatry 4, no. 10 (October 2017): 749–758, 10.1016/S2215-0366(17)30328-0. 28888927 PMC5614772

