# Supplementary material for: Couples Sharing Sleep and Sickness: Dyadic Sleep‐Wake Patterns, Health Consequences, and Intervention Efficacy in Cancer Patient‐Caregiver Dyads—A Systematic Review and Preliminary Meta‐Analysis
Source: Psychooncology. 2026 May 11;35:e70480. doi: 10.1002/pon.70480 (PMC13159513; doi:10.1002/pon.70480)
Supplement: Supplementary file 2 — Supporting Information S2 [file PON-35-e70480-s001.docx]

**JBI Checklist for Analytical Cross-Sectional Studies**

|  | Diamantis (2025) | Ellis (2024) | Tsai (2024) | Ting (2024) | Vachon (2024) | Salomo (2024) | Kwekkeboom (2024) | Schuler (2023) | Fenech (2022) | Perndorfer (2022) |
| --- | --- | --- | --- | --- | --- | --- | --- | --- | --- | --- |
| Were the criteria for inclusion in the sample clearly defined? | Y | Y | Y | Y | Y | Unclear (no clear exclusion criteria) | Y | Y | Y | Y |
| Were the study subjects and the setting described in detail? | Y | Y | Y | Y | Y | Y | Y | Y | Y | Y |
| Was the exposure measured in a valid and reliable way? | Y | Y | Y | Y | Y | Y | Y | Y | Y | Y |
| Were objective, standard criteria used for measurement of the condition? | Y | Y | Y | Y | Y | Y | Y | Y | Y | Y |
| Were confounding factors identified? | Y | Y | Y | Y | Y | Y | Y | Not applicable (feasibility test of ecological momentary assessment) | Y | Y |
| Were strategies to deal with confounding factors stated? | Y | Y | Y | Y | Y | Y | Y | Not applicable | Y | Y |
| Were the outcomes measured in a valid and reliable way? | Y | Y | Y | Y | Y | Y | Y | Y | Y | Y |
| Was appropriate statistical analysis used? | Y | Y | Y | Y | Y | Y | Y | Y | Y | Y |

Yes/No/Unclear/Not applicable

|  | He (2022) | Ellis (2021) | Chen (2020) | Otto (2019) | Chan (2017) | Kotronoulas (2016) | Hsiao (2014) | Carney (2011) | Gibbins (2009) |
| --- | --- | --- | --- | --- | --- | --- | --- | --- | --- |
| Were the criteria for inclusion in the sample clearly defined? | Y | Y | Y | Y | Unclear (no exclusion criteria) | Y | Y | Y | Y |
| Were the study subjects and the setting described in detail? | Y | Y | Y | Y | Y | Y | Y | Y | Y |
| Was the exposure measured in a valid and reliable way? | Y | Y | Y | Y | Y | Y | Y | Y | Y |
| Were objective, standard criteria used for measurement of the condition? | Y | Y | Y | Y | Y | Y | Y | Y | Y |
| Were confounding factors identified? | Y | Y | Y | Y | Y | Y | Y | Y | Y |
| Were strategies to deal with confounding factors stated? | Y | Y | Y | Y | Y | Y | Y | Y | Y |
| Were the outcomes measured in a valid and reliable way? | Y | Y | Y | Y | Y | Y | Y | Y | Y |
| Was appropriate statistical analysis used? | Y | Y | Y | Y | Y | Y | Y | Y | Y |

Yes/No/Unclear/Not applicable

**Cochrane Risk of Bias (RoB V2)**

|  | Randomization process | Deviations from intended interventions (including blinding of interventionist and contamination) | Measurement of outcomes (blinding of outcome assessors) | Missing outcome data (attrition bias) | Selection of reported results |
| --- | --- | --- | --- | --- | --- |
| Mosher (2024) | Low | Low | Some concerns (blinding not reported) | Low | Low |
| Burns (2023) | Low | Low | Low | Low | Low |
| Hsiao (2016) | Low | Low | Some concerns (blinding not reported) | Low | Low |

Low risk of bias, Some concerns, High risk of bias

**Risk of Bias in Non-Randomized Studies of Interventions, Version 2 (ROBINS-I V2)**

|  | Confounding | Selection of participants into the study | Classification of interventions | Deviations from intended interventions | Missing data | Measurement of the outcome | Selection of the reported result |
| --- | --- | --- | --- | --- | --- | --- | --- |
| Kim (2024) | Moderate (No control of time-varying covariates) | Low | Low | Low | Serious (High attrition rate after intervention) | Low | Low |
| Rhudy (2023) | Moderate (No control of time-varying covariates) | Low | Low | Low | Serious (High attrition rate at Week 12 after intervention) | Low | Low |
| Johns (2020) | Moderate (No control of time varying covariates) | Low | Low | Low | Low | Low | Low |
| Milbury (2018) | Moderate (No control of time-varying covariates) | Low | Low | Low | Serious (High attrition rate post-intervention) | Low | Low |
| Milbury (2015b) | Moderate (No control of time-varying covariates) | Low | Low | Low | Moderate (High attrition rate but the dyads withdrew did not differ from study completers) | Low | Low |
| Milbury (2015a) | Moderate (No control of time-varying covariates) | Low | Low | Low | Moderate (High attrition rate but the dyads withdrew did not differ from study completers) | Low | Low |

Low risk of bias, Moderate risk of bias, Serious risk of bias, and Critical risk of bias
